# Pathologic Roles of Receptor-Associated Prorenin System in Idiopathic Epiretinal Membrane

**DOI:** 10.1038/srep44266

**Published:** 2017-03-09

**Authors:** Yoko Dong, Atsuhiro Kanda, Kousuke Noda, Wataru Saito, Susumu Ishida

**Affiliations:** 1 Laboratory of Ocular Cell Biology and Visual Science, Hokkaido University Graduate School of Medicine, Sapporo, Hokkaido, 060-8638, Japan; 2 Department of Ophthalmology, Hokkaido University Graduate School of Medicine, Sapporo, Hokkaido, 060-8638, Japan

## Abstract

Receptor-associated prorenin system (RAPS) refers to the pathogenic mechanism whereby prorenin binding to (pro)renin receptor [(P)RR] dually activates tissue renin-angiotensin system (RAS) and RAS-independent signaling via (P)RR. The aim of this study is to determine the association of RAPS with idiopathic epiretinal membrane (iERM). Reverse transcription-PCR indicated the expression of RAPS components, including (P)RR and Ang II type 1 receptor (AT1R), in iERM tissues and human Müller glial cell line. Double-labeling analyses demonstrated that (P)RR and AT1R were detected in cells positive for glial fibrillary acidic protein, a marker for glial cells, and co-localized with prorenin and angiotensinogen, respectively. Administration of prorenin to Müller glial cells enhanced mRNA expression of *fibroblast growth factor 2*, while Ang II application stimulated the expression of *glial cell line-derived neurotrophic factor, nerve growth factor*, and *transforming growth factor-β1*. These expression levels induced by prorenin or Ang II were reversed by (P)RR or AT1R blockade, respectively. Immunofluorescence revealed tissue co-localization of (P)RR and AT1R with the products of the upregulated genes *in vitro*. The present findings suggest the involvement of RAPS in the pathogenesis of iERM.

Idiopathic epiretinal membrane (iERM) is a leading cause of visual acuity loss, metamorphopsia, micropsia, and occasionally monocular diplopia in the elderly, although it varies in severity with cases asymptomatic or suffering from impaired central visual function despite good visual acuity^1^. The prevalence rate of iERM in those aged 50 or older was recently estimated to be 7.7%, which is higher than that of early age-related macular degeneration (6.8%)^2^,^3^. The development of iERM basically requires the remnant of posterior vitreous cortex on the surface of the posterior retina as well as the migration and proliferation of Müller glial cells, both of which are triggered by posterior vitreous detachment^4^,^5^,^6^,^7^. The pathogenesis of iERM also involves multiple cytokine responses related to its classically known fibrotic processes^8^; however, its molecular mechanism still remains largely unknown.

The renin-angiotensin system (RAS), a known key regulator of systemic blood pressure and water balance (circulatory RAS), contributes to vascular inflammation in various organs (tissue RAS) including the retina^9^. Tissue RAS is initiated by prorenin binding with (pro)renin receptor [(P)RR] to acquire renin activity, which also causes RAS-independent signal transduction in cells bearing (P)RR. This dual activation is called the receptor-associated prorenin system (RAPS), which has been shown to play significant roles in the molecular pathogenesis of retinal disorders such as inflammation and pathologic angiogenesis^10^,^11^. Moreover, we recently reported that the protein levels of prorenin and soluble (P)RR increased in the vitreous fluids obtained from patients with proliferative diabetic retinopathy (PDR)^12^,^13^. Interestingly, Maruichi *et al*. measured the intravitreal enzymatic activity of chymase and angiotensin-converting enzyme (ACE), both of which are related to angiotensin II (Ang II) generation, and revealed that chymase and ACE were activated in idiopathic macular diseases (i.e., macular hole and ERM) and PDR, respectively^14^. This led us to hypothesize that chymase-induced RAS activation may be involved in the pathogenesis of iERM.

In this study, we examined localization and expression of RAPS components in surgically excised iERM tissues, together with molecular mechanisms causing fibrosis-related reactions in Müller glial cells, so as to define the role of RAPS in the pathogenesis of iERM.

## Results

### Expression of RAPS Components in iERM Tissues and Müller Glial Cells

**Figure 1:**
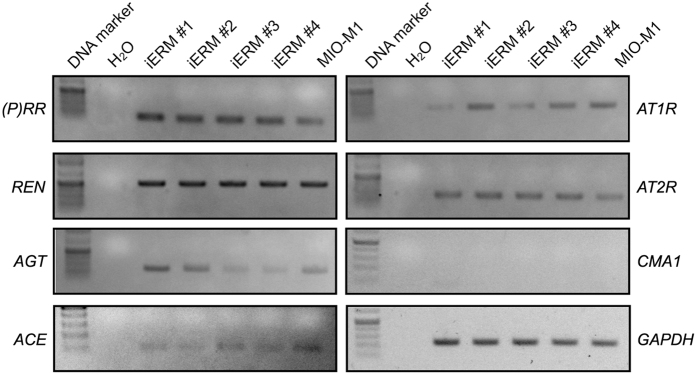
Expression of RAPS components in iERM tissues and Müller cells. RT-PCR analysis was performed to check the expression of RAPS components [(*P)RR, REN, AGT, ACE, AT1R*, and *AT2R*] and *CMA1* in four iERM tissues (iERM #1–4) and MIO-M1 cells. *GAPDH* was used as an internal control.

To identify the pathological role of RAPS in iERM, we examined the gene expression of RAPS components [(*P)RR, prorenin (REN*), *angiotensinogen (AGT*), *ACE, Ang II type 1 receptor (AT1R*) and *AT2R*] and *chymase 1 (CMA1*) in iERM tissues and human Müller glial cell line (MIO-M1). Reverse transcription-PCR (RT-PCR) analyses showed that RAPS components were expressed in iERM tissues and human Müller glial cell line MIO-M1, suggesting the potential involvement of prorenin-(P)RR and Ang II-AT1R axes in the pathogenesis of iERM. *CMA1* expression was detected in human retinas and placentas (Supplementary Fig. S1), but not in iERM tissues (Fig. 1).

### Localization of Prorenin and (P)RR in iERM Tissues

**Figure 2:**
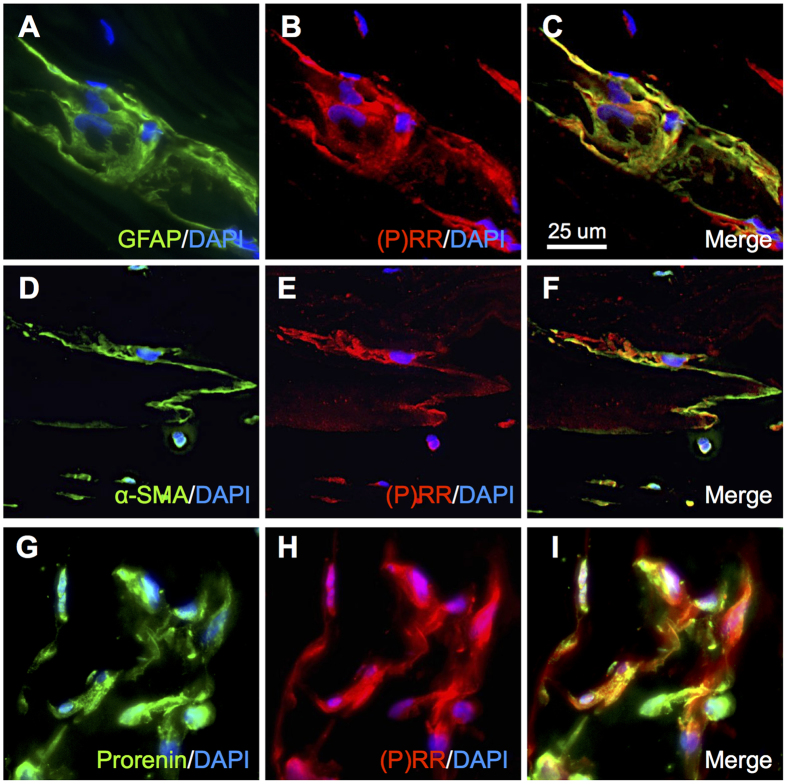
Localization of prorenin and (P)RR in iERM tissues. (**A**–**C**) Double labeling of GFAP (*green*), (P)RR (*red*) and DAPI (*blue*). (**D**–**F**) Double labeling of α-SMA (*green*), (P)RR (*red*) and DAPI (*blue*). (**G**–**I**) Double labeling of prorenin (*green*), (P)RR (*red*) and DAPI (*blue*). Scale bar: 25 μm.

To validate the gene expression results, we performed immunofluorescence analysis to examine the localization of (P)RR in iERM tissues. According to previous reports, Müller glial cells and myofibroblasts constituting iERM tissues were positive for glial fibrillary acid protein (GFAP) and α-smooth muscle actin (α-SMA), respectively^8^,^15^,^16^. Double labeling experiments revealed co-localization of (P)RR signal with GFAP, a glial cell marker (Fig. 2A–C) and α-SMA, a myofibroblast marker (Fig. 2D–F). Moreover, protein expression of (P)RR in glial cells abundantly co-localized with prorenin (Fig. 2G–I).

### Localization of AGT and AT1R in iERM Tissues

**Figure 3:**
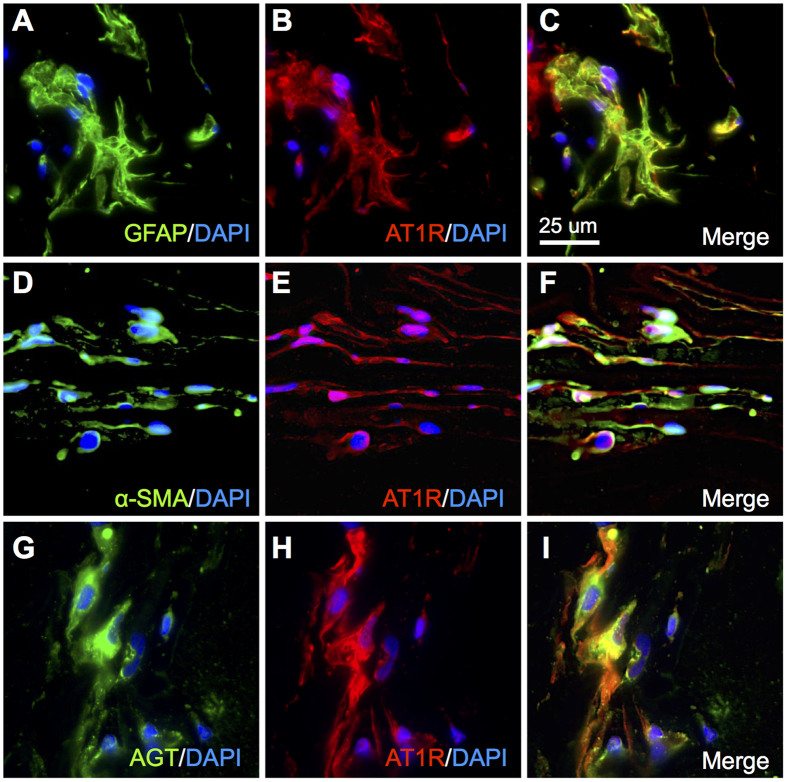
Localization of AGT and AT1R in iERM tissues. (**A**–**C**) Double-labeling of GFAP (*green*), AT1R (*red*) and DAPI (*blue*). (**D**–**F**) Double-labeling of α-SMA (*green*), AT1R (*red*) and DAPI (*blue*). (**G**–**I**) Double-labeling of AGT (*green*), AT1R (*red*) and DAPI (*blue*). Scale bar: 25 μm.

To further study the localization of AT1R in iERM tissues, we carried out double labeling of AT1R with GFAP and α-SMA. AT1R co-localized with both GFAP and α-SMA, indicating the expression of AT1R in glial cells (Fig. 3A–C) and myofibroblasts (Fig. 3D–F), respectively, in iERM tissues. In addition, AT1R immunoreactivity also co-localized with AGT in glial cells of iERM tissues (Fig. 3G–I).

### Localization of ACE and AT2R in iERM Tissues

**Figure 4:**
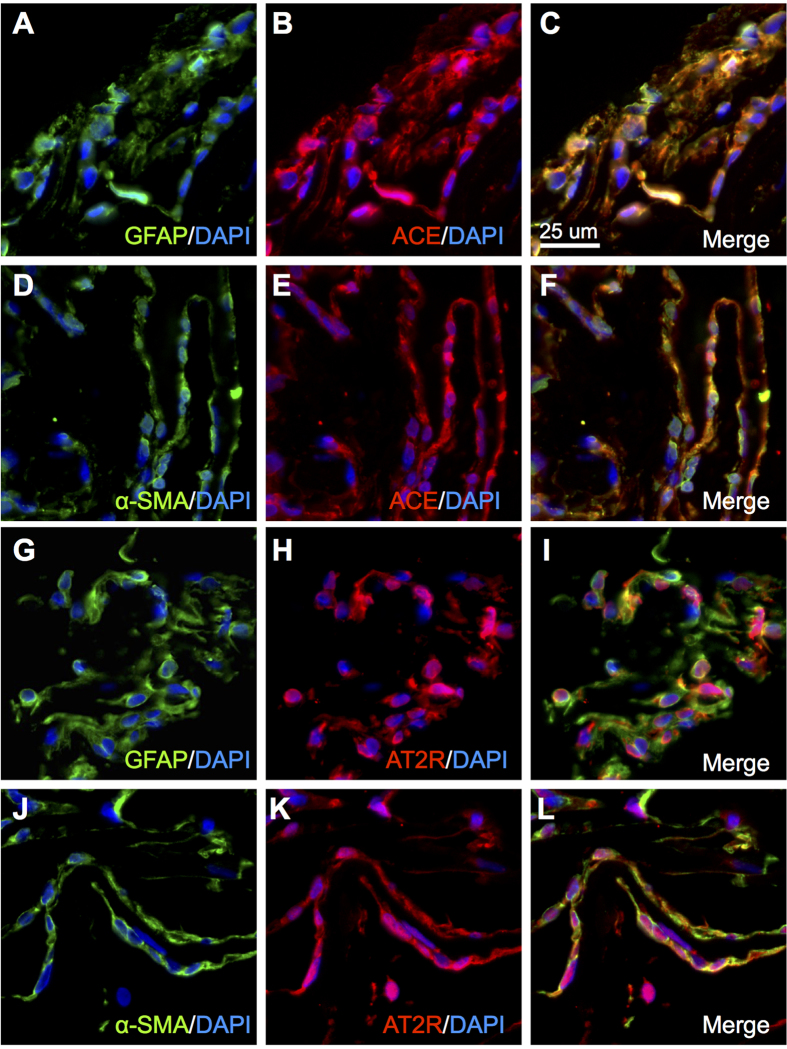
Localization of ACE and AT2R in iERM tissues. (**A**–**C**) Double-labeling of GFAP (*green*), ACE (*red*) and DAPI (*blue*). (**D**–**F**) Double-labeling of α-SMA (*green*), ACE (*red*) and DAPI (*blue*). (**G**–**I**) Double-labeling of GFAP (*green*), AT2R (*red*) and DAPI (*blue*). (**J**–**L**) Double-labeling of α-SMA (*green*), AT2R (*red*) and DAPI (*blue*). Scale bar: 25 μm.

Next, we checked immunoreactivity for the remaining RAPS components whose gene expression was detected in iERM tissues (Fig. 1). ACE signals were strongly positive for GFAP (Fig. 4A–C) and α-SMA (Fig. 4D–F), suggesting the generation of Ang II in iERM tissues also containing AGT, prorenin and (P)RR proteins (Figs 2 and 3), all of which are required for tissue RAS activation in concert with ACE^10^,^11^.

**Figure 5:**
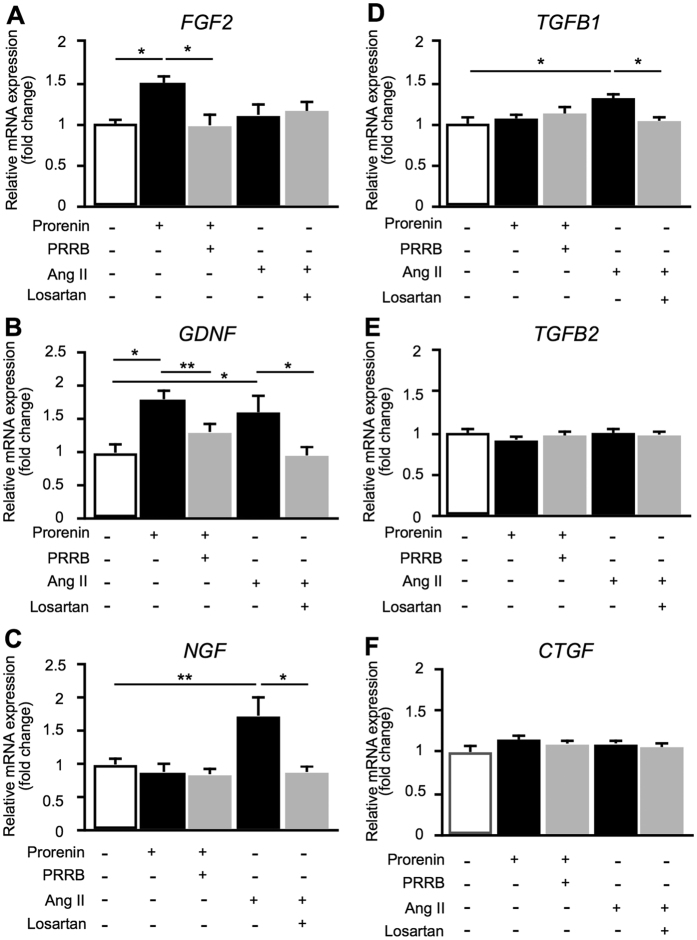
Upregulation of fibrosis-related cytokines by prorenin and Ang II in Müller cells. (**A**–**F**) Relative RNA expression levels of *FGF2, GDNF, NGF, TGFB1, TGFB2*, and *CTGF* in MIO-M1 cells stimulated by prorenin or Ang II with or without those receptor antagonists (n = 8 per group). *p < 0.05, **p < 0.01.

AT2R is known to antagonize the AT1R-mediated functions including cell growth and inflammation through competitive interaction with Ang II^17^. In accordance with this, AT2R protein expression in cells positive for GFAP (Fig. 4G–I) and α-SMA (Fig. 4J–L) was equivalent with AT1R signals (Fig. 3). Since Ang II-induced pathogenesis depends mainly on AT1R but not on AT2R, we focused on AT1R on top of (P)RR in the following *in vitro* inhibition experiments (Fig. 5).

### Upregulation of Fibrosis-Related Cytokines by Prorenin and Ang II in Müller Glial Cells

Prorenin-(P)RR and Ang II-AT1R axes have been shown to induce the expression of several genes both *in vivo* and *in vitro*, and contribute to the pathogenesis of numerous ocular diseases^9^,^10^,^11^,^12^. Histological analyses of iERM tissues indicated a variety of cells including glial cells, hyalocytes, fibroblasts and myofibroblasts^18^,^19^,^20^. Among them, Müller glial cells, a main constituent cell type in iERM, are the most important cellular components required for membrane growth by intracellular signal transduction^15^,^19^. Several studies described the involvement of cytokines and trophic factors in the pathogenesis of iERM^4^,^8^,^16^,^21^,^22^,^23^. Stimulation of fibroblasts with Ang II are known to promote collagen synthesis^24^; however, little is known about whether this is the case with Müller glial cells.

To determine the effects of prorenin and Ang II on Müller glial cells, we studied mRNA expression levels of fibrosis-related cytokines in human Müller glial cell line MIO-M1 by real-time quantitative PCR (qPCR) analysis. Prorenin stimulation to cells significantly increased the expression levels of *fibroblast growth factor* 2 (*FGF2*) (fold change = 1.49, *p* < 0.05) and *glial cell line-derived neurotrophic factor (GDNF*) (fold change = 1.81, *p* < 0.01) mRNA levels compared to those of controls, while pretreatment of human (P)RR blocker (PRRB) suppressed prorenin-induced *FGF2* and *GDNF* expression (*FGF2*, fold change = 0.99; *GDNF*, fold change = 1.31) (Fig. 5A,B). Interestingly, increased *GDNF* expression was also eliminated by AT1R blocker losartan (Supplementary Fig. S2A), suggesting that *GDNF* expression was driven by RAS signaling pathway, but not (P)RR-induced intracellular signal transduction.

On the other hand, administration of Ang II to MIO-M1 cells significantly increased the expression of *GDNF* (fold change = 1.61, *p* < 0.05), *nerve growth factor (NGF*) (fold change = 1.74, *p* < 0.01) and *transforming growth factor-β1 (TGFB1*) (fold change = 1.31, *p* < 0.05) compared to controls (Fig. 5B–D). Importantly, increased *GDNF, NGF* and *TGFB1* expression levels were reduced by pretreatment with losartan (*GDNF*, fold change = 0.96; *NGF*, fold change = 0.89; *TGFB1*, fold change = 1.04). *NGF* and *TGFB1* were also upregulated by prorenin stimulation with a longer duration of 48 hours, and again suppressed by losartan (Supplementary Fig. S2B,C), suggesting that prorenin acquired renin activity through interaction with (P)RR causing tissue RAS activation and subsequent AT1R-mediated expression of *NGF* and *TGFB1*.

In contrast, there were no significant differences in the expression levels of *TGFB2* and *connective tissue growth factor (CTGF*) mRNA following either prorenin or Ang II treatment (Fig. 5E,F).

### Co-localization of Prorenin- and Ang II-Induced Cytokines with (P)RR and AT1R in iERM Tissues

**Figure 6:**
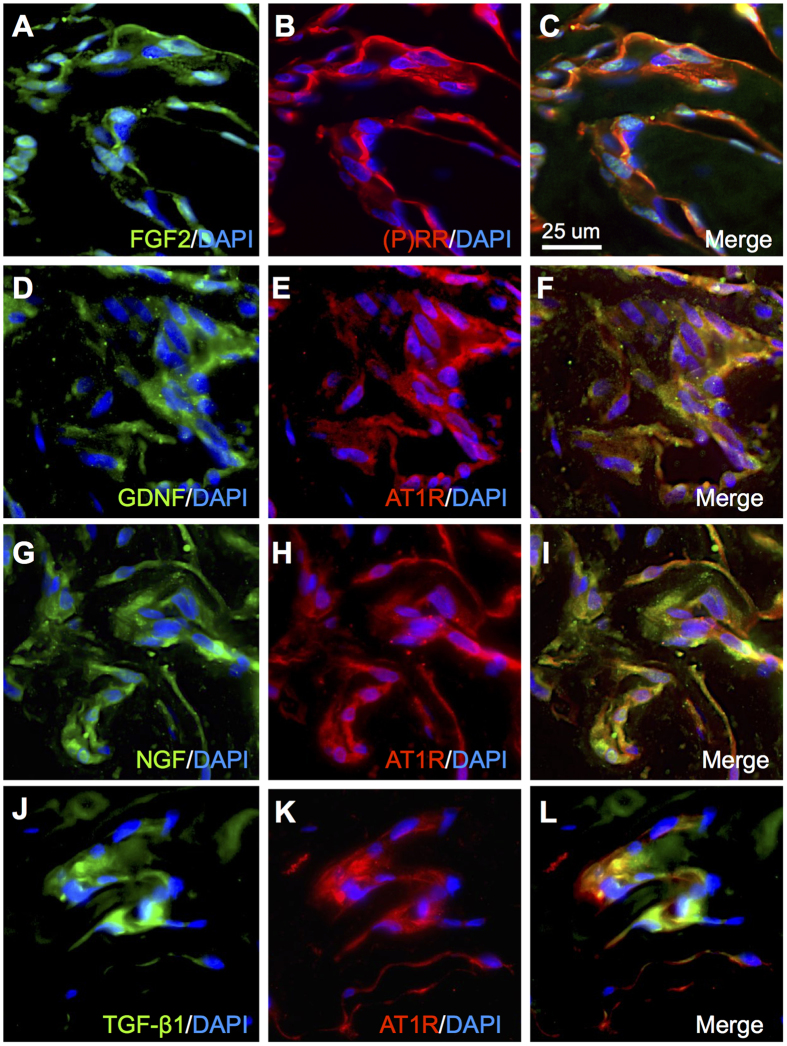
Co-localization of prorenin- and Ang II-induced cytokines with (P)RR and AT1R in iERM tissues. (**A**–**C**) Double labeling of FGF2 (*green*), (P)RR (*red*) and DAPI (*blue*). (**D**–**F**) Double labeling of GDNF (*green*), AT1R (*red*) and DAPI (*blue*). (**G**–**I**) Double-labeling of NGF (*green*), AT1R (*red*) and DAPI (*blue*). (**J**–**L**) Double labeling of TGF-β1 (*green*), AT1R (*red*) and DAPI (*blue*). Scale bar: 25 μm.

Based on *in vitro* gene expression results, we performed immunofluorescence analysis to examine the co-localization of FGF2, GDNF, NGF, and TGF-β1 with (P)RR and AT1R in iERM tissues. Immunofluorescence analysis revealed that FGF2 immunoreactivity co-localized with (P)RR in iERM tissues (Fig. 6A–C). In addition, GDNF, NGF and TGF-β1 signals co-localized with AT1R (Fig. 6D–L). These results suggest that the activation of RAPS, using both (P)RR- and AT1R-downstream pathways, induces pro-fibrotic cytokine expression in Müller cells and contributes to the pathogenesis of iERM.

## Discussion

The present study demonstrated, for the first time to our knowledge, the involvement of RAPS in the pathogenesis of iERM. First, transcripts of RAPS components were expressed in surgically removed iERM tissues and human Müller glial cell line (Fig. 1). (P)RR and AT1R immunoreactivity co-localized with prorenin and AGT in iERM tissues, respectively (Figs 2 and 3). ACE signals were also immuno-positive in iERM, suggesting the generation of Ang II in iERM tissues (Fig. 4). Stimulation with prorenin to Müller glial cells increased the expression of *FGF2* through (P)RR activation, while Ang II induced the expression of *GDNF, NGF* and *TGFB1* through AT1R activation (Fig. 5). Prorenin-induced *GDNF, NGF* and *TGFB1* expression levels were reversed by AT1R blocker losartan (Supplementary Fig. S2), verifying the pivotal role of (P)RR to trigger tissue RAS activation leading consequently to AT1R signal transduction. Immunofluorescence analyses showed co-localization of (P)RR and AT1R with the corresponding pro-fibrotic cytokines in iERM tissues (Fig. 6).

Both AT1R and AT2R were detected in GFAP and α-SMA positive cells of iERM tissues (Figs 3 and 4). AT2R, another cognate receptor for Ang II, is dominantly expressed in the fetus, while its expression increases in the pathophysiologic conditions such as vascular injury and cardiac remodeling in the adult. Ang II-AT1R axis mainly mediates the Ang II-induced pathogenesis, whereas AT2R counteracts the AT1R-mediated pathophysiological events (hypertension, cell growth, inflammation, etc.) due to competitive interaction with their ligand Ang II and direct inhibition of AT1R dimerization required for its signaling^17^. Although the effects mediated by AT2R remain to be fully clarified, AT2R is likely to negatively regulate pathological changes in iERM tissues.

**Figure 7:**
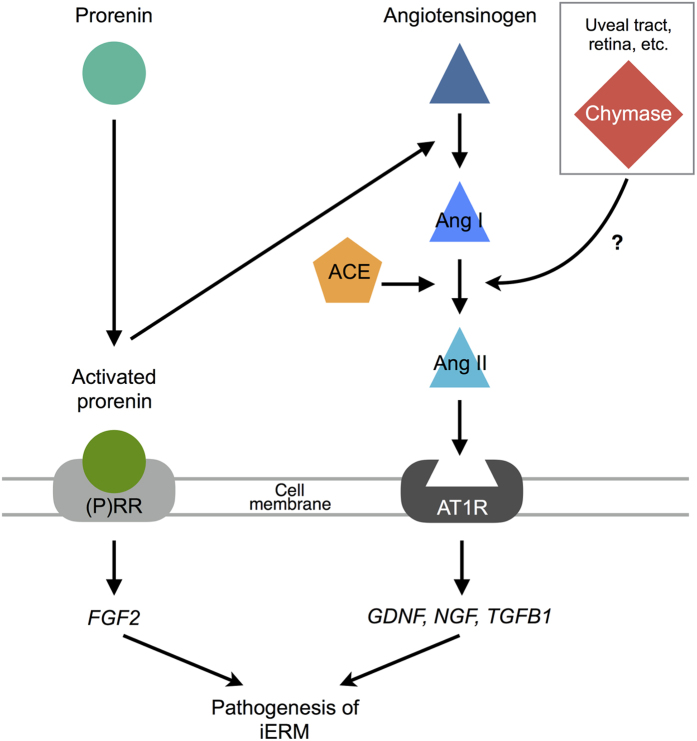
A schema showing the significant involvement of (P)RR and AT1R in the pathogenesis of iERM (modified from Kanda *et al*.^13^).

Several cytokines and trophic factors were detected in iERM tissues and vitreous fluids, suggesting that the activation of signal transduction in glial cells by those molecules leads to iERM formation and progression^4^,^8^,^16^,^21^,^22^. FGF2, a member of a large family of neurotrophic molecules, supports survival and maturation of glial cells^21^,^25^. GDNF, a member of the TGF-β-related neurotrophic factor family, increases FGF2 production in glial cells^25^. NGF and TGF-β1 have been reported to stimulate glial cells to transdifferentiate into myofibroblasts^16^,^22^. Our current data showed that the expression of these fibrosis-related molecules in iERM were governed by RAPS-mediated mechanisms (Fig. 7).

So far, we have revealed the association of RAPS with ocular inflammation and neovasucularization in animal disease models and human clinical samples^9^,^10^,^11^,^12^,^13^,^26^. In patients with PDR, surgically excised fibrovascular tissues demonstrated the localization of RAPS components, leading to vascular endothelial growth factor (VEGF)-driven angiogenesis via both (P)RR and AT1R signaling pathways^9^,^12^,^13^. Given that fibrovascular proliferation consists of both fibrosis and angiogenesis, the currently observed role of RAPS in fibrous (i.e., non-angiogenic) proliferation suggests its additional contribution to fibrosis, on top of angiogenesis, in the pathogenesis of PDR. Indeed, previous reports revealed the significant role of RAPS in promoting fibrosis in the heart and kidney^27^,^28^. Further and future studies are waited for to clarify the possible involvement of RAPS with other fibrotic disorders in the eye including proliferative vitreoretinopathy and PDR.

Chymase, a serine protease produced mainly by mast cells, converts Ang I to Ang II. The vitreous harbors two Ang II-generating systems: chymase and ACE that were shown to preferentially function in eyes with iERM and PDR, respectively^14^. The ACE enzymatic activity in the vitreous of iERM eyes was quite low^14^, although our current analysis detected the gene and protein expression of *ACE* in all the iERM tissues examined (Figs 1 and 4). In contrast, we have recently demonstrated the expression of *ACE* in fibrovascular tissues extracted from eyes with PDR^12^, in accordance with the previous data showing the high ACE activity in the vitreous of PDR eyes^14^, whereas our present observation failed to detect *CMA1* expression in iERM tissues (Fig. 1). In mammalian eyes, the chymase enzymatic activity was detected mainly in the uveal tract^29^, while we demonstrated *CMA1* gene expression in the human retina (Supplementary Fig. S1). Taken together, chymase in iERM eyes is thought to be supplied from regions (uveal tract, retina, etc.) other than the lesion per se, unlike ACE in PDR eyes, and contribute to the generation of Ang II in parallel with ACE (Fig. 7).

However, the vitreous levels of Ang II were significantly lower in eyes with idiopathic macular diseases (i.e., ERM and macular hole) than in PDR eyes, suggesting the mild to moderate activation of RAS in the vitreous of eyes with idiopathic macular diseases^30^. This is explained by and consistent with our recent data showing the elevated levels of soluble (P)RR protein^12^ as well as renin activity^13^ in the vitreous of PDR compared to idiopathic macular diseases. These findings may indicate the substantially lower activation of RAPS in iERM than in PDR, which would reasonably reflect a difference in pathogenic activity between fibrosis alone and fibrosis plus angiogenesis. Nevertheless, our present data may not only lead to a new understanding of the molecular pathogenesis of iERM but may also allow for subsequent development of new pharmacologic therapies for preventing the development and deterioration of this disease.

## Methods

### Human Surgical Samples

iERM tissues were collected from 10 eyes of 10 patients, who underwent pars plana vitrectomy for iERM. Six iERM tissues were used for immunofluorescence analyses, and another 4 iERM tissues were processed for gene expression analyses. The clinical characteristics of the patients in this study are listed in Table 1. This study was conducted in accordance with the tenets of the Declaration of Helsinki and after receiving approval from the institutional review board of Hokkaido University Hospital. All patients gave written informed consent after our explanation of the purpose and procedures of this study (IRB #015-0226). We obtained human (healthy adults) retina and placenta cDNAs as a kind gift from Dr. Anand Swaroop (National Eye Institute, Bethesda, MD, USA).

### Cell Culture and Chemicals

The human Müller glial cell line (MIO-M1) was provided from Dr. G. Astrid Limb (UCL Institute of Ophthalmology, London, United Kingdom)^31^. The cells were cultured in Dulbecco’s modified Eagle’s medium containing 10% fetal bovine serum (Life Technologies, Carlsbad, CA, USA). To cover the handle region of the prorenin molecule, which is the binding site of (P)RR^12^, decoy peptides NH_2_-RIFLKRMPSI-COOH as human (P)RR blocker (PRRB) were synthesized and purified using high-pressure liquid chromatography on a C-18 reverse-phase column by GeneDesign (Osaka, Japan). The purity and retention time of HPLC was 96.4% and 16.7 minutes, respectively. The mass of the product was 1261.0 and similar to the theoretical mass value (1260.6). After the cells were serum deprived, MIO-M1 cells were pretreated with 1 μM PRRB or 10 μM AT1R blocker losartan (Sigma-Aldrich, St. Louis, MO, USA) for 1 hour. Prorenin or Ang II was then added at a final concentration of 10 nM or 1 μM, respectively. Cells were incubated for 24 hours and processed for analysis to detect RNA expression levels.

### Reverse Transcription-PCR (RT-PCR) and Real-Time Quantitative PCR (qPCR) Analysis

Total RNA isolation and reverse transcription were performed from cells using SuperPrep Cell Lysis & RT Kit for qPCR (TOYOBO, Tokyo, Japan) with oligo dT and random primers following the manufacturers’ protocols, and from tissues using TRIzol (Life Technologies) and GoScrip Reverse Transcriptase (Promega, Madison, WI, USA) with oligo dT(20) primers, as previously described^12^. The following primers for genes were used: *FGF2* (forward 5′-ACG GCG TCC GGG AGA A-3′, reverse 5′-ACA CTC CCT TGA TGG ACA CAA CT-3′), *GDNF* (forward 5′-CCA ACC CAG AGA ATT CCAGA-3′, reverse 5′-AGC CGC TGC AGT ACC TAA AA-3′), *NGF* (forward 5′-ATA CAG GCG GAA CCA CAC TC-3′, reverse 5′-TGC TCC TGT GAG TCC TGT TG-3′), *TGFB1* (forward 5′-GCC CTG GAC ACC AAC TATT G-3′, reverse 5′-CGT GTC CAG GCT CCA AAT G-3′), *TGFB2* (forward 5′-AGA GTG CCT GAA CAA CGG ATT-3′, reverse 5′-CCA TTC GCC TTC TGC TCT T-3′), *CTGF* (forward 5′-TTG GCA GGC TGA TTT CTA GG-3′, reverse 5′-GGT GCA AAC ATG TAA CTT TTG G-3′), *CMA1* (forward 5′-AAC ACT TCT ACT CTT CAC CACGA-3′, reverse 5′-GGC TTC AAC ACA CCT GTT CTT-3′), and *glyceraldehyde-3-phosphate dehydrogenase (GAPDH;* forward 5′-AGG TCG GTG TGA ACG GAT TTG-3′, reverse 5′-TGT AGA CCA TGT AGT TGA GGT CA-3′). RAPS-related genes were analyzed as described in our previous report^12^. Real-time qPCR was performed using the GoTaq qPCR Master mix (Promega) and StepOne plus Systems (Life Technologies).

### Immunofluorescence Microscopy

Paraffin sections of iERM tissues were deparaffinized and hydrated through exposure with xylene and graded alcohols followed by water. As a pretreatment, microwave-based antigen retrieval was performed in 10 mM citrate buffer (pH 6). Sections were incubated with the following primary antibodies: rabbit anti-(P)RR (Sigma-Aldrich), mouse anti-prorenin, rabbit anti-ACE and rabbit anti-AT2R (Abcam, Cambrige, MA, USA), rabbit anti-AT1R and goat anti-AGT (Santa Cruz Biotechnology, Santa Cruz, CA, USA), mouse anti-FGF2 (Millipore, Temecula, CA, USA), goat anti-GDNF, goat anti-NGF, mouse anti-TGF-β1 and mouse anti-α-SMA (R&D systems, Minneapolis, MN, USA), and mouse anti-GFAP (Leica, Exton, PA, USA) antibodies. Secondary antibodies for fluorescent detection were AlexaFluor 488 and 546 (Life Technologies). Sections were examined using the Keyence BZ-9000 (Keyence, Osaka, Japan).

### Statistical Analysis

All the results are expressed as the mean ± SEM. Student’s t-test following the analysis of variance (ANOVA) was used for statistical comparison between groups. Differences between means were considered statistically significant when *p* values were <0.05.

## Additional Information

**How to cite this article:** Dong, Y. *et al*. Pathologic Roles of Receptor-Associated Prorenin System in Idiopathic Epiretinal Membrane. *Sci. Rep.* **7**, 44266; doi: 10.1038/srep44266 (2017).

**Publisher's note:** Springer Nature remains neutral with regard to jurisdictional claims in published maps and institutional affiliations.

## Supplementary Material

Supplementary Figures

## Figures and Tables

**Table 1:** Clinical characteristics of patients with iERM.

Case	Age (years)	R/L	Sex	History of ocular surgery	Decimal visual acuity
1	85	R	F	Cataract (5 years ago)	0.4
2	69	L	F	None	0.7
3	64	L	F	None	0.7
4	68	R	M	None	0.6
5	75	L	F	None	0.4
6	62	R	F	None	0.4
7	75	R	M	Cataract (5 years ago)	0.6
8	76	L	F	None	0.9
9	70	R	M	None	0.5
10	52	L	F	None	0.5
