# Supplementary material for: Pathologic Roles of Receptor-Associated Prorenin System in Idiopathic Epiretinal Membrane
Source: Sci Rep. 2017 Mar 9;7:44266. doi: 10.1038/srep44266 (PMC5343583; doi:10.1038/srep44266)
Supplement: Supplementary Figures [file srep44266-s1.pdf]

Pathologic Roles of Receptor-Associated Prorenin System in Idiopathic Epiretinal Membrane

Yoko Dong, Atsuhiro Kanda, Kousuke Noda, Wataru Saito and Susumu Ishida

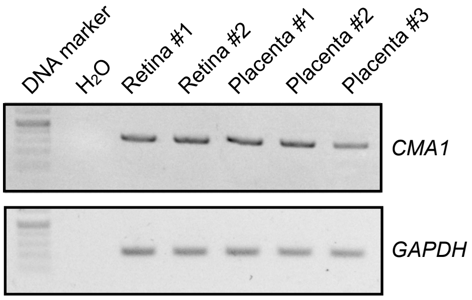

Supplementary Figure S1. Gene expression of *CMA1* in human retinas and placentas. RT-PCR analysis was performed to check the expression of *CMA1* in two retinas and three placentas.

Pathologic Roles of Receptor-Associated Prorenin System in Idiopathic Epiretinal Membrane

Yoko Dong, Atsuhiro Kanda, Kousuke Noda, Wataru Saito and Susumu Ishida

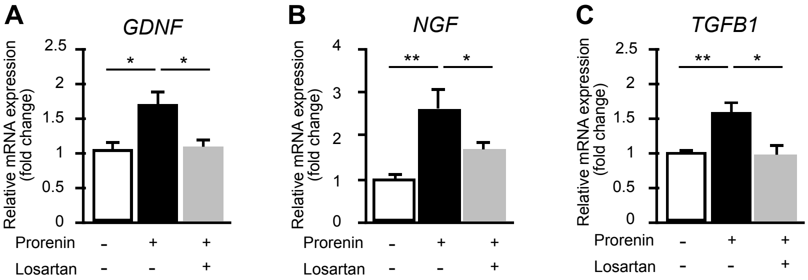

Supplementary Figure S2. (P)RR-mediated upregulation of *GDNF, NGF* and *TGFB1* in MIO-M1 cells. A, Relative mRNA expression levels of *GDNF* in prorenin-stimulated MIO-M1 cells with/without losartan for 24h (n = 8 per group). **B-C,** Relative mRNA expression levels of *NGF* and *TGFB1* in MIO-M1 cells stimulated by prorenin with/without losartan for 48h (n = 8 per group). *$p < 0.05$, **$p < 0.01$.
